# Type XXVIII Collagen Regulates Renal Interstitial Fibrosis and Epithelial-Mesenchymal Transition by SREBP1-Mediated HKDC1 Expression

**DOI:** 10.1155/2022/9582559

**Published:** 2022-11-26

**Authors:** Linlin Li, Qi Zou, Binbin Li, Lushi Huang, Lixin Wei

**Affiliations:** Department of Nephrology, Fujian Medical University Union Hospital, Fujian, Fuzhou 350001, China

## Abstract

**Background:**

A novel collagen called type XXVIII collagen (COL28) is involved in cancer and lung fibrosis. Preliminary data showed that renal tubular epithelial cells could proliferate, migrate, and undergo an epithelial-mesenchymal transition (EMT) when COL28 was overexpressed; however, it is still unknown how this occurs and what the underlying mechanism is.

**Methods:**

We analyzed the differential expression of genes (DEGs) in the stable COL28 overexpression HK-2 cell lines by RNA-sequencing analysis, before which Gene Ontology (GO) and Kyoto Encyclopedia of Gene and Genomes (KEGG) analyses were performed. Genes related to COL28 promoting HK-2 cell proliferation and EMT were screened and verified. By using western blot and immunofluorescence, the effects of COL28 on the expression of *α*-SMA, E-cadherin, Snail, HKDC1, and SREBP1 were detected. The effect of COL28 overexpression on renal fibrosis in unilateral ureteral obstruction (UUO) mice was detected by H&E and Masson staining. HKDC1 interference agent was synthesized and transfected into the HK-2 cell line stably overexpressing COL28. In HK-2 cells, the effects of HKDC1 interference on the expression of *α*-SMA, E-cadherin, and Snail were detected.

**Results:**

We screened and verified that HKDC1 was related to COL28 and promoted HK-2 cell proliferation and EMT. WB showed that in HK-2 cells, COL28 overexpression increased *α*-SMA, Snail, HKDC1, and SREBP1 expressions and decreased E-cadherin expression. Overexpression of COL28 aggravated renal interstitial fibrosis in UUO mice; upregulated *α*-SMA, Snail, HKDC1, and SREBP1 expressions; and decreased the E-cadherin protein expression in UUO mice. Interference of HKDC1 expression promoted the E-cadherin protein expression while inhibiting *α*-SMA, Snail, HKDC1, and SREBP1 protein expressions.

**Conclusion:**

Overexpression of COL28 can aggravate renal interstitial fibrosis by encouraging renal tubular epithelial cells to undergo EMT, and interference with HKDC1 expression can alleviate fibrosis by reversing EMT induced by COL28 overexpression.

## 1. Introduction

Chronic kidney disease (CKD) is a worldwide health issue with a significant and increasing death toll [1]. The gradual progression of CKD can lead to renal failure [2]. Tubulointerstitial fibrosis (TIF), which possesses the histopathologic characteristics of inflammatory cell infiltration, extracellular matrix (ECM) accumulation, peritubular microvasculature loss, and tubular atrophy, is the hallmark of CKD [3]. The renal tubular epithelial-to-mesenchymal transition (EMT), secretion of a large number of ECM components and deposition in the extracellular matrix, is a key link in the aggravation of renal interstitial fibrosis [4].

Collagens, a large family of triple helical proteins, are found all over the body and are crucial for a variety of processes [5]. Collagens were previously thought to play a structural role, but more recent research has demonstrated that they also play a significant role in a broad range of physiological processes, including cell proliferation, adhesion, survival, and migration [6]. Collagen XXVIII, one of a total of 28 kinds of collagens, has only recently been described, and not much information is available about its precise function. It has two von Willebrand domains located next to a 528-amino acid collagenous domain, giving it a structure that is similar to type VI collagen [7].

Collagen XXVIIIL, encoded by the COL28A1 gene located on mouse chromosome 6A1 and human chromosome 7p21.3, is limitedly expressed in tissues. Except for type II terminal Schwann cells in the hairy skin, all nonmyelinating glial cells and dorsal root ganglia are surrounded by collagen XXVIIIL which is primarily present in the peripheral nerves [8, 9]. Recent research indicated that COL28 is involved in cancer and lung fibrosis [7, 9]. For instance, type XXVIII collagen was reported to express at extremely low levels in healthy lung tissues; however, the overexpression of type XXVIII collagen in bleomycin-induced lung injury was revealed by Schiller et al., characterized by intense staining with a patchy appearance in fibrotic foci [10]. They also used coimmunostaining to confirm that different cell lineages secreted and assembled collagen XXVIII in response to injury. Our previous research suggested that COL28 expression was significantly increased in fibrotic renal tissues [11]. Overexpressed COL28 may aggravate renal fibrosis by encouraging renal tubular epithelial cells (HK-2) to proliferate, differentiate, and accelerate their EMT processes, but the exact mechanism is waiting for exploration.

High-throughput RNA-sequencing (RNA-seq), also known as transcriptome sequencing, is a method for studying transcriptomics based on second-generation sequencing technology [12]. It can measure all mRNA and noncoding RNA sequence information transcribed by any cell in a certain functional state [13, 14]. We sequenced the transcriptome of the HK-2 cell line stably transfected with COL28 by RNA-seq technology, analyzed the transcriptome expression profile of the differentially expressed genes (DEGs), and screened the DEGs related to the overexpression of COL28. Then, Kyoto Encyclopedia of Gene and Genomes (KEGG) pathway and Gene Ontology (GO) enrichment analyses were employed to analyze the function of the related DEGs. Combining the role of COL28 in promoting the proliferation, differentiation, and EMT of HK-2 cells in previous studies as well as with bioinformatics analysis results and literature review, we found that HKDC1 was a DEG related to COL28 overexpression.

Hexokinase (HK), a rate-limiting enzyme converting glucose into glucose-6-phosphate, regulates glucose metabolism in various organisms. The fifth hexokinase to be recently identified is hexokinase domain component 1 (HKDC1). The HKDC1 gene is located on human chromosome 10 q22. The protein expression is mainly located in the duodenum, small intestine, thyroid, kidney, bladder, and other parts [15, 16]. Following a 2-hour glucose tolerance test on women who were 28 weeks pregnant, a genome-wide association research discovered HKDC1 to be related to gestational blood glucose levels [17]. Metabolic disorders are critical in the onset and progression of malignancies, and HKDC1 plays an important part in this by catalyzing glucose phosphorylation. Therefore, some researchers have studied the relationship between HKDC1 and tumors. The results revealed the possibility of HKDC1 as a new prospective target for tumor treatment [18]. Zhang et al. studied the relationship between HKDC1 and liver cancer and found that HKDC1 expression level was notably increased in liver cancer tissues, with a higher HKDC1expression level related to a worse prognosis. Hepatoma cell proliferation and migration can be inhibited by silencing the HKDC1 *in vitro* expression [19]. Chen et al. discovered an increased HKDC1 expression level in breast tumor tissues, which could effectively promote cell proliferation, while knockdown of HKDC1 inhibited cell proliferation [20]. In addition, the HKDC1 overexpression enhanced the cell invasion and migration.

We hypothesized that interference with HKDC1 expression could alleviate fibrosis by reversing EMT induced by COL28 overexpression. To prove our hypothesis, we investigated how COL28 overexpression affected renal fibrosis in UUO mice. Further studies were performed to clarify the relevant mechanism between HKDC1 and COL28 gene involved in cell fibrosis.

## 2. Materials and Methods

### 2.1. Cell Culture

HK-2 cells (human kidney proximal tubular cell line) were bought from the American Type Culture Collection (Manassas, VA, USA). The cells were cultured in Dulbecco's modified Eagle's medium/F12 medium (HyClone, USA), which contains 10% FBS (HyClone, USA) and 1% antibiotics (100 U/mL penicillin and 100 *μ*g/mL streptomycin) (Life Technologies Carlsbad, CA). In an incubator that was humidified and contained 5% CO_2_, the cells were incubated at 37°C. For the TGF-*β*1-treated experiment, HK-2 cells were grown in a serum-free medium with 10 ng/mL recombinant human TGF-*β*1 (PeproTech, CA#10021 catalog, USA).

### 2.2. Construction of COL28A1 Overexpressed Lentivirus and Adenovirus

The COL28A1-expressing lentivirus vector (Lv-COL28A1), the negative universal control (Lv-NC), the COL28A1-expressing adenovirus vector (Av-COL28A1), and the negative universal control (Av-NC) containing the green fluorescent protein EGFP and a puromycin resistance gene were established by Shanghai Genechem Co., Ltd. (Shanghai, China). COL28 overexpressed lentivirus was used in cell experiments, and COL28 overexpressed adenovirus was used in animal experiments.

### 2.3. Cell Transfection

Before infection with the virus, the HK-2 cells were inoculated into a 6-well plate (1.0 × 10^5^ cells/well) and allowed to grow to reach 30% confluence. The lentiviruses Lv-COL28A1 and Lv-NC were then transfected into the cells with an enhanced infection solution (Shanghai Genechem Co., Ltd.) and polybrene (Sigma, USA). Puromycin (3 *μ*g/mL) was added to select the transfected HK-2 cells, and then, the expression of the fluorescence protein EGFP was used to monitor the infection efficiency by fluorescence microscope after 72 h. The Lv-COL28A1 positive cells were designated as COL28-OE, and the Lv-NC cells were designated as COL28-NC.

siRNA, specifically targeting HKDC1, was transiently transfected into HK-2 cells. Han Heng Biological Co., Ltd. supplied SiHKDC1 and si-Control (negative reference), which were then diluted in Opti medium until the concentration reached 80 nM. Lipo 3000 (Thermo Fisher, USA) was chosen as the transfection reagent, which was mixed with siRNA and given to the cells after 15 minutes of incubation. Following 8 hours of incubation, the transfected cells were then subjected to various stimuli after the medium was withdrawn.

### 2.4. RNA-seq

The cells were assigned to one of the three groups: control group, COL28 negative control group (COL28-NC group), and COL28 overexpression group (COL28-OE group). The extraction, library building, and sequencing of total RNA of cells were performed by Aimer Gene (Xiamen) Biotechnology Co., Ltd. TRIzol (Invitrogen) was employed to extract the total RNA from the samples, NanoDrop 2000 (Thermo Fisher Scientific) to evaluate the quality and concentration of the RNA, and QIAxcel advanced system (Qiagen, advanced) to assess RNA integrity. With the help of NEB NextUltra™ RNA Library Prep Kit for Illumina (New England BioLabs, Inc., Ipswich, MA, USA), RNA-seq libraries were created under the recommendations of the manufacturer. Using the Illumina HiSeq 4000 instrument (Illumina, Inc., San Diego, CA, USA), the quality-checked libraries were sequenced, and paired-end reads were produced. Initial processing of the raw data in FASTQ format was done with an internal Perl script. By removing low-quality reads, ploy-N, and reads containing adapters, the clean reads were achieved. For the purpose of assessing the sequencing quality, the GC content and Q20 were determined. Utilizing star (for genome comparison), cufflinks (for expression analysis), and deseq2 (for differential expression), the obtained reads were analyzed [21].

### 2.5. Identification of DEGs

Fragments per kilobase of exon per million fragments mapped (FPKM) were used to determine gene expression values. By means of DESeq with cutoff values of fold change ≥ 2 and false discovery rate (FDR) < 0.01, DEGs between the COL28-OE group and the COL28-NC group were identified.

### 2.6. Functional Enrichment Analysis of DEGs

GO, which covers three subcategories, cellular component (CC), biological process (BP), and molecular function (MF), has annotated the functions of DEGs. In order to identify the signaling pathways where the DEGs were concentrated, KEGG pathway analysis was carried out. With a threshold of FDR < 0.05, GO and KEGG enrichment analyses were done on the Database for Annotation, Visualization and Integrated Discovery (DAVID).

### 2.7. Animal Model

C57BL/6J male mice weighing 25–30 g at 8 weeks old and in the male gender were purchased from SLAC Laboratory Animal Co. (Hunan, China). Five experimental groups of mice were generated: control group, merely undergoing anesthesia (inhalation anesthesia with 2% isoflurane) and laparotomy, and UUO + COL28-NC group, submitted to anesthesia (inhalation anesthesia with 2% isoflurane) and 100 *μ*L of empty adenovirus (1.58 × 10^10^ pfu/mL) through renal vein injection. Then, the animal underwent left unilateral ureteral occlusion 7 days after renal intravenous injection; in the Sham operate + COL28-NC group, the animal was submitted to anesthesia (inhalation anesthesia with 2% isoflurane) and 100 *μ*L of empty adenovirus (1.58 × 10^10^ pfu/mL) through renal intravenous injection. Then, the animal was submitted to a laparotomy 7 days after renal vein injection; in the UUO + COL28-OE group, the animal was submitted to anesthesia (inhalation anesthesia with 2% isoflurane) and 100 *μ*L of COL28 overexpression adenovirus (1.58 × 10^10^ pfu/mL) through renal vein injection. The animal was then submitted to left unilateral ureteral occlusion for 7 days after renal vein injection; in the Sham operate + COL28-OE group, the animal was submitted to anesthesia (inhalation anesthesia with 2% isoflurane) and 100 *μ*L of COL28 overexpression adenovirus (1.58 × 10^10^ pfu/mL) through renal vein injection. Then, the animal was submitted to a laparotomy 7 days after renal vein injection. All animals were sacrificed by cervical dislocation after inhalation anesthesia with 2% isoflurane on the 14th day after renal vein injection. The kidney tissue was collected for subsequent experiments. The Animal Ethics Committee of Fujian Medical University gave its approval to all animal experiments.

### 2.8. Quantitative Real-Time PCR (qRT-PCR)

The TRIzol reagent (Invitrogen, Carlsbad, CA, USA) was applied to isolate the total RNA and the high-capacity cDNA Reverse Transcriptase Kit (Roche, Mannheim, Germany) to transcribe the two micrograms of RNA reversely. In Table 1, a list of qRT-PCR primers is provided. The SYBR Green PCR Master Mix Kit (Invitrogen, Carlsbad, CA, USA) was adopted to accomplish qRT-PCR amplification. The cycling conditions were as follows: initial denaturation was performed for 3 minutes at 95°C, followed by three temperature PCR (denaturing, 95°C for 30 seconds; annealing, 57.5°C for 30 seconds; and extension, 72°C for 30 seconds) in 35 cycles, and a final extension for 10 minutes at 72°C was done, after which the temperature was lowered to 4°C. *β*-Actin was adopted to normalize the relative quantity of mRNA, which was then calculated by the delta-delta method from threshold cycle numbers. Using the 2^−∆∆Ct^ method, the amount of the amplified molecules at the threshold cycle was assessed based on exponential amplification of the target gene and calibrator.

### 2.9. Western Blot

Following cell lysis in cell lysis buffer (Beyotime, Shanghai, China) containing 1% PMSF, the cells were quantified using a BCA Protein Assay Kit (Beyotime). A total of 30 *μ*g of protein were transferred to nitrocellulose membranes after being run through an 8%-15% SDS-PAGE. The membrane was first blocked for 1 hour at ambient temperature with PBS containing 5% nonfat dry milk and then incubated with the following primary antibodies overnight at 4°C: rabbit anti-collagen XXVIII (1 : 1000; ab188533, Abcam, Cambridge, MA, USA), mouse anti-E-cadherin (1 : 1000; Ab76055, Abcam), mouse anti-alpha smooth muscle actin (1 : 1000; GB13044, Servicebio, Wuhan, China), and mouse anti-GAPDH (1 : 1000; AF7021, Affinity Biosciences, Melbourne, Australian). Later, the membranes were treated with horseradish peroxidase-conjugated anti-rabbit or mouse IgG (1 : 5000; Jackon, Immuno Research, USA) after three 10-minute washings in PBS buffer containing 0.05% Tween 20. Following the final washing, an enhanced chemiluminescence reagent (Millipore, Bedford, MA, USA) was supplemented to develop the membranes, and the specific protein bands related to *β*-actin or GADDH were scanned and quantitated. The images were analyzed for optical density using the ImageJ software.

### 2.10. Immunofluorescence

Prior to permeabilization with 0.1% Triton X-100 in PBS, the cells were fixed with 4% paraformaldehyde at ambient temperature for 20 min. Primary antibodies mouse anti-alpha smooth muscle actin (1 : 200; GB13044, Servicebio, Wuhan, China) and mouse anti-E-cadherin (1 : 200; ab76055, Abcam) were supplied for the overnight incubation at 4°C of the samples which were previously blocked with 5% bovine serum albumin in PBS. The samples were washed three times in PBS before being incubated for 1 hour at ambient temperature with goat anti-mouse IgG Cy3 conjugated (1 : 200; CWS0145S, CWBIO). Upon being shook dry, the samples were stained with a DAPI staining solution for 15 min. The addition of antifluorescence quenching agent was following soon after. Under fluorescence microscope, the cells were observed in different channels. For the acquisition of the relative fluorescence intensity, the ImageJ was used.

### 2.11. Histopathological and Masson Examinations

For histopathological examination, the kidney tissue was stored in a 10% neutral buffered formaldehyde solution for 24 h before being embedded in paraffin. Subsequent to being cut into 3 *μ*m, the sections were dewaxed with xylene and stained with hematoxylin and eosin (H&E) and Masson trichrome.

### 2.12. Statistical Analysis

SPSS version 21.0 was applied to analyze the data from this experiment. The data are displayed as mean ± standard deviation (SD). The statistical analysis for comparing various treatment and control groups was carried out using a two-way analysis of variance (ANOVA) followed by Dunnett's test. We used the Student *t*-test to compare the differences between the two groups. *P*values below 0.05 were statistically significant.

## 3. Result

### 3.1. Identification of DEGs in COL28 Overexpressing HK-2 Cells

**Figure 1:**
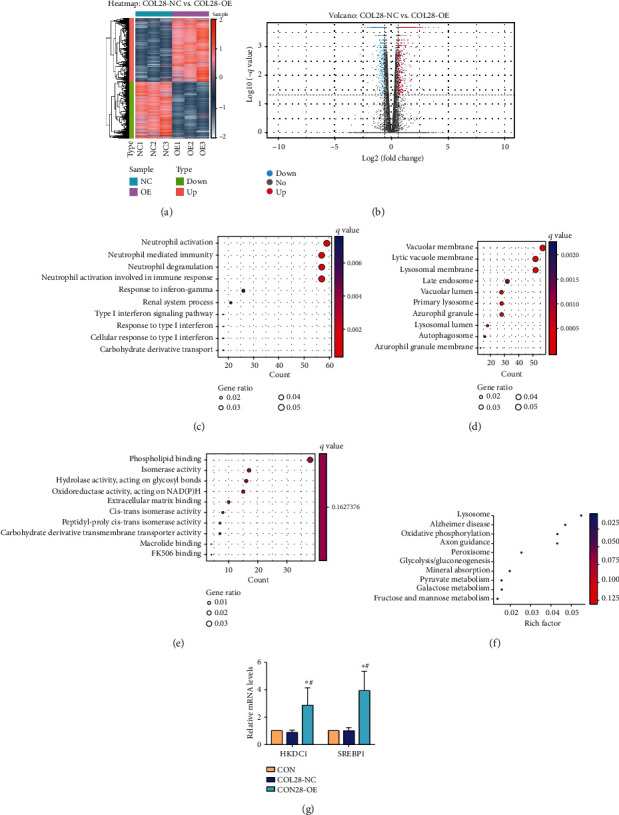
Strategy of RNA-seq analysis and identification of DEGs in the COL28-NC group and COL28-OE group in HK-2 cells. (a) Heatmap of differentially expressed mRNAs in the COL28-NC and COL28-OE groups. Red denotes upregulated genes, while blue indicates downregulated genes. (b) Volcano map of the COL28-NC group and COL28-OE group's differentially expressed genes in HK-2 cells. (c–e) Gene Ontology analysis enrichment of the number distribution position of differential genes. (f) Kyoto Encyclopedia of Genes and Gene Pathways (KEGG) analyzes metabolic pathways considerably enriched in differentially expressed genes in the COL28-NC and COL28-OE groups. (g) Identification of HKDC1 and SREBP1 gene expression by qRT-PCR.

A total of 444 DEGs were screened, among which 286 were significantly differentially upregulated and 158 were significantly differentially downregulated (Figures 1(a) and 1(b)). The top fifteen high/low expression genes between the COL28-OE and COL28-NC groups are listed in Tables 2 and 3.

GO analysis (Figures 1(c)–1(e)) showed that relative to the COL28-NC group, the top 3 response processes of HK-2 after overexpressing the COL28 gene in BP were primarily connected to neutrophil activation and neutrophil degranulation involved in immune responses. Compared with the COL28-NC group, the top three highly expressed DEGS in CC in the COL28-OE group are vacuolar membrane, dissolved vacuolar membrane, and lysosomal membrane. Compared with the COL28-NC group, the top three highly expressed DEGS in MF in the COL28-OE group were phospholipid binding, isomerase activity, and hydrolase activity.

Next, in order to further clarify the role of COL28, we carried out KEGG pathway enrichment analysis. The main biological pathways in DEGs were lysosome, oxidative phosphorylation, Alzheimer's disease, galactose metabolism, glycolysis and glycogenesis, mineral absorption, peroxisome, pyruvate metabolic disorder, axon orientation, fructose, and mannose metabolism (Figure 1(f)).

We searched the top 30 DEGs in the COL28-OE group and the top 10 pathways on the KEGG pathway enrichment bubble chart alternately, combined with the fact that the proliferation ability of HK-2 cells overexpressing COL28 increased, and searched relevant literature. We found that HKDC1 was related to the proliferation of the overexpressed COL28 gene. We verified the HKDC1 expression level in the COL28-OE group by qRT-PCR and observed that the COL28-OE group had a significantly higher HKDC1 gene expression level than the CON group and COL28-NC group (*P* < 0.05) (Figure 1(g)). Furthermore, the COL28-OE group showed a higher SREBP1 expression level than the COL28-NC group (*P* < 0.05).

### 3.2. Overexpression of COL28 Promotes EMT in HK-2 Cell

**Figure 2:**
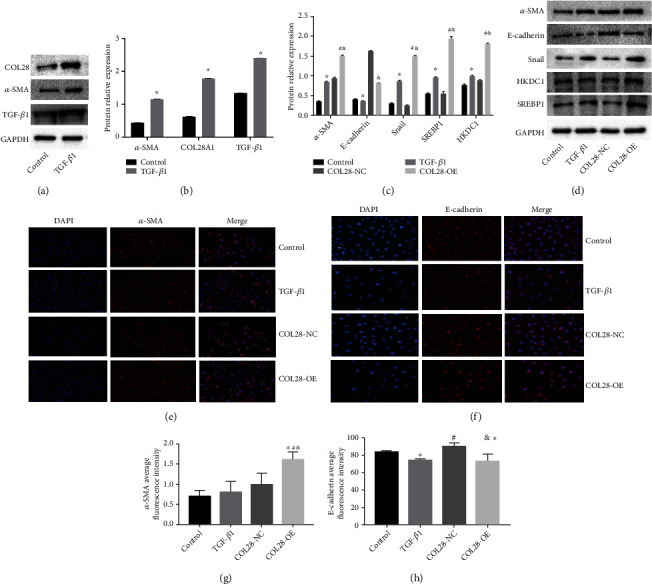
Overexpression of COL28 promotes EMT in HK-2 cells. (a, b) 10 ng/mL TGF-*β*1 promotes HK-2 cell *α*-SMA, COL28, and TGF-*β*1 expression (^∗^*P* < 0.05 vs. control, *n* = 3). (c, d) The effect of COL28 overexpression in HK-2 cells on the *α*-SMA, E-cadherin, Snail, HKDC1, and SREBP1 expressions by WB (^∗^*P* < 0.05 vs. control, ^#^*P* < 0.05 vs. TGF-*β*1, ^&^*P* < 0.05 vs. COL28-NC, *n* = 3). (e–h) Overexpression of COL28 on the EMT of HK-2 cells detected by immunofluorescence (^∗^*P* < 0.05 vs. control, ^#^*P* < 0.05 vs. TGF-*β*1, and ^&^*P* < 0.05 vs. COL28-NC).

We used 10 ng/mL TGF-*β*1 to stimulate the HK-2 cells. The COL28A1 expression, -smooth muscle actin (-SMA), and TGF-1 in HK-2 cells were determined by western blotting (WB). It can be seen that TGF-*β*1 significantly promoted COL28A1, *α*-SMA, and TGF-*β*1 expressions in HK-2 cells (Figures 2(a) and 2(b)).

In contrast to the control group, 10 ng/mL of TGF-*β*1 increased the *α*-SMA expression and decreased the E-cadherin expression in HK-2 cells; that is, TGF-*β*1 could promote EMT in HK-2 cells. Meanwhile, Snail, HKDC1, and SREBP1 expressions could be increased. Compared with the COL28-NC group, COL28A1 overexpression significantly reduced E-cadherin expression in HK-2 cells and significantly increased *α*-SMA, Snail, SREBP1, and HKDC1 expressions (Figures 2(c) and 2(d)).

Compared with the COL28-NC group, COL28A1 overexpression increased the *α*-SMA immunofluorescence expression and significantly reduced the E-cadherin immunofluorescence expression in HK-2 cells (*P* < 0.05, Figures 2(e)–2(h)).

### 3.3. Effect of COL28 Overexpression on Renal Fibrosis in UUO Mice

**Figure 3:**
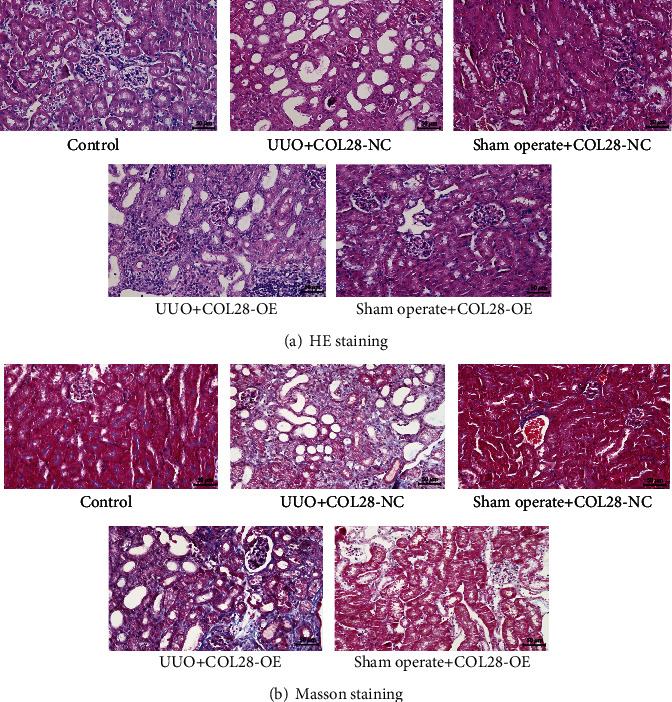
Effects of renal vein injection of COL28 overexpression adenovirus on renal histopathology in UUO mice. (a) The effect of COL28 overexpression on renal histopathology in UUO mice detected by H&E staining. (b) The effect of COL28 overexpression on renal histopathology in UUO mice detected by Masson staining.

We constructed an adenovirus overexpressing COL28 and injected it into the renal tissue of UUO mice through their renal vein to detect the effect of COL28 overexpression on renal fibrosis in UUO mice. As can be seen from Figure 3(a), contrasted with the control group and the Sham operation group, the renal tubular epithelial cells in the UUO + COL28-NC group became flat, necrotic, and exfoliated, the lumen was significantly enlarged, inflammatory cells infiltrated the interstitium, and the tissue structure was disorganized, suggesting that the UUO mouse model was successfully constructed. By comparison with the UUO + COL28-NC group, the UUO+COL28 overexpression group had obvious infiltration of inflammatory cells in the stroma and aggravated renal fibrosis. As can be seen from the Masson staining in Figure 3(b), the area dyed blue in the UUO + COL28 overexpression group was significantly elevated relative to the UUO + COL28-NC group, suggesting that the degree of renal fibrosis was aggravated; that is, the overexpression of COL28 in the renal tissue of UUO mice could aggravate renal interstitial fibrosis.

### 3.4. Effects of COL28 Overexpression Adenovirus Pretreatment on Renal Fibrosis in UUO Mice

**Figure 4:**
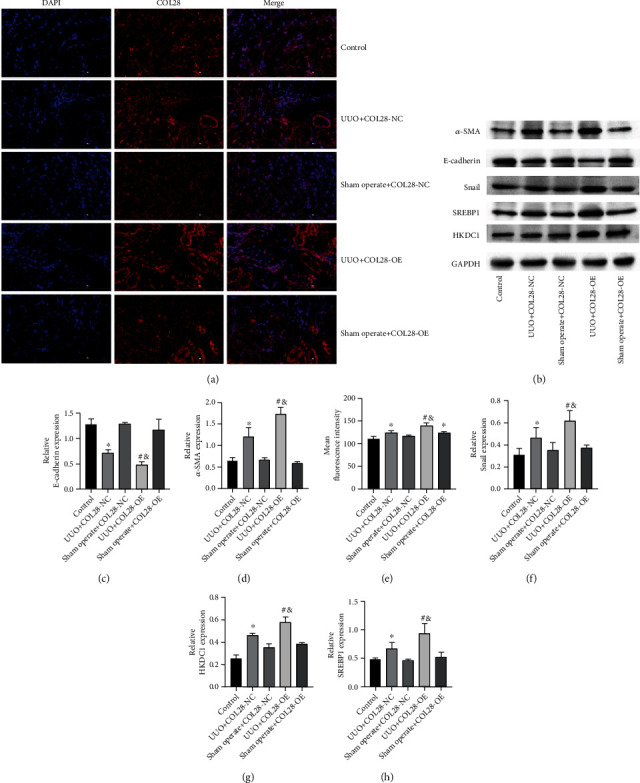
Effects of COL28 overexpression adenovirus pretreatment on renal fibrosis in UUO mice. (a, e) Expression of COL28 in the kidneys of UUO mice pretreated with COL28 overexpression adenovirus determined by immunofluorescence assay. (b–d, f–h) *α*-SMA, E-cadherin, Snail, HKDC1, and SREBP1 expressions in the kidney of UUO mice pretreated with COL28 overexpression adenovirus were determined by WB (^∗^*P* < 0.05 vs. control, ^#^*P* < 0.05 vs. UUO+COL28-NC, ^&^*P* < 0.05 vs. Sham operate+COL28-OE, *n* = 3).

In Figure 4, in comparison to the control group, we can see that the COL28 immunofluorescence expression in the UUO+COL28-NC group was significantly increased (Figure 4(a)), with a statistical significance (Figure 4(e)), confirming that the fibrosis of obstructive nephropathy could promote the increase of endogenous COL28 expression. The UUO+COL28-OE group presented a further enhanced COL28 fluorescence expression than the UUO+COL28-NC group, suggesting that the pretreatment of COL28 adenovirus was successful and COL28 could be effectively overexpressed in the UUO model (Figures 4(a) and 4(e)).

It can be seen that after UUO modeling, *α*-SMA and Snail expressions obviously upregulated (*P* < 0.05), and E-cadherin expression apparently downregulated (*P* < 0.05). In the UUO+COL28OE group, the *α*-SMA and Snail expressions were elevated (*P* < 0.05), while the E-cadherin expression was further reduced (*P* < 0.05). These results suggest that COL28 can promote renal fibrosis induced by UUO, which may be achieved by promoting EMT. Relative to the control group, the UUO group showed higher HKDC1 and SREBP1 expressions in renal tissues (*P* < 0.05), while the overexpression of COL28A1 could further promote the expressions of HKDC1 and SREBP1. Combined with the RNA-seq and *in vitro* studies results, HKDC1 was shown to be the downstream regulatory point of COL28 promoting EMT (Figures 4(b)–4(d) and 4(f)–4(h)).

### 3.5. Effects of HKDC1 Interference on EMT Protein Expression in HK-2 Cells with Stable COL28A1 Overexpression

**Figure 5:**
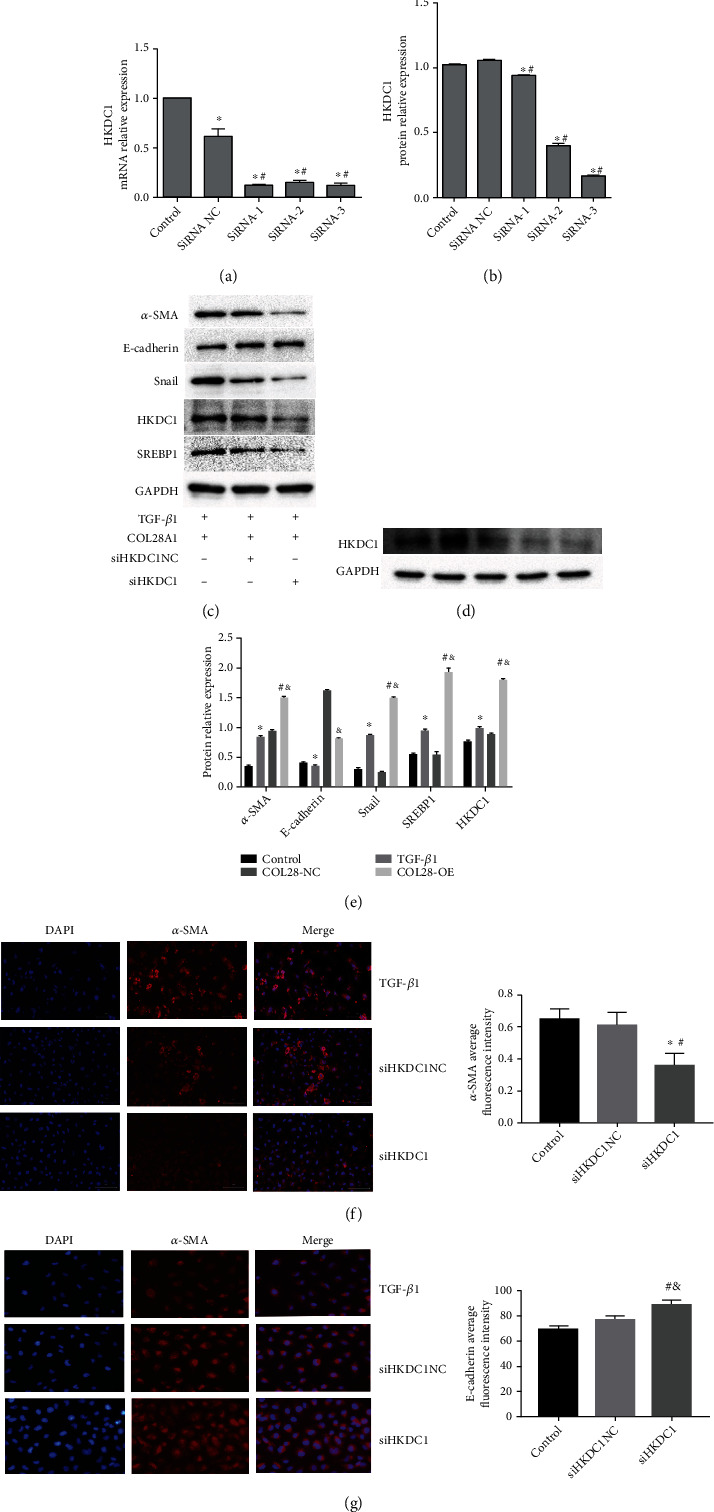
HKDC1 knockdown may enhance EMT in HK-2 cells stimulated by COL28 overexpression. (a, b) HKDC1 interference was successfully generated and stably transfected into HK-2 cells. HKDC1 interference efficiency was evaluated by qPCR and WB. ^∗^*P* < 0.05 vs. control, ^#^*P* < 0.05 vs. siRNA NC, *n* = 3. (d, e) Influence of HKDC1 intervention on the expression levels of *α*-SMA, E-cadherin, Snail, SREBP1, and HKDC1 detected by WB. ^∗^*P* < 0.05 vs. model, ^#^*P* < 0.05 vs. siHKDC1, *n* = 3. (f, g) Expression levels of *α*-SMA and E-cadherin by immunofluorescence. ^∗^*P* < 0.05 vs. control, ^&^*P* < 0.05*vs*. siHKDC1 NC, *n* = 3.

Three HKDC1 interference vectors were designed and transfected into the stable HK-2 cell line overexpressed by COL28A1. The HKDC1 expression level was detected by qPCR and WB (Figures 5(a)–5(c)). siRNA-3 had the best interference effect, which was selected for subsequent experiments. WB was used to detect the influence of HKDC1 intervention on the expression levels of *α*-SMA, E-cadherin, Snail, SREBP1, and HKDC1. The results are shown in Figures 5(d) and 5(e). It can be seen that the expression levels of *α*-SMA, Snail, SREBP1, and HKDC1 were markedly reduced after HKDC1 interference (*P* < 0.05) and E-cadherin was considerably elevated in HK-2 cells with stable COL28A1 overexpression (*P* < 0.05). The results are shown in Figures 5(f) and 5(g). With respect to the siHKDC1 NC group, the siHKDC1 group significantly reduced the *α*-SMA immunofluorescence expression and increased E-cadherin the immunofluorescence expression in HK-2 cells (*P* < 0.05). HKDC1 interference can reverse the EMT induced by COL28A1 overexpression.

## 4. Discussion

Collagens are a superfamily present in every tissue in the vertebrate body and crucial for maintaining tissue integrity [6]. Besides the most important structural role, collagens' function also includes cell adhesion, proliferation, migration, and survival [22, 23]. A novel member of the collagen family, collagen XXVIII is a kind of the VWA domain-containing branch protein. It is located in the endocrine tissues, proximal digestive tract, central nervous system, lung, kidney, etc. Recent studies have suggested that COL28 is involved in cancer and lung fibrosis. Yang et al. screened 13 key genes related to the prognosis of glioblastoma multiforme, among which COL28A1 was the most important [24]. COL28 is involved in lung fibrosis and might be a therapeutic target [10]. Polymorphisms and mutations in COL28 might be involved in kidney fibrosis [11], but the exact mechanism of COL28 in renal fibrosis is unknown.

Our preliminary data showed that the COL28 expression in renal fibrosis was high in both human and mouse models. Studies conducted *in vitro* revealed that COL28 overexpression stimulated renal tubular epithelial cells to proliferate, migrate, and undergo EMT. In the current research, we confirmed again that TGF-*β*1 could promote the increase of COL28 expression, while overexpression of COL28 could promote the upregulation of *α*-SMA expression and the downregulation of E-cadherin expression.

Further, we constructed an adenovirus for COL28 overexpression, pretreated it to mouse kidneys by renal vein injection, and then established an obstructive nephropathy model by ligating unilateral ureter to find how COL28 overexpression influenced renal fibrosis in UUO mice. H&E staining showed that the renal inflammatory cells in the UUO+COL28-OE group infiltrated significantly, and the tissue structure was more disordered, while Masson staining showed an increase in fibrotic area, relative to the UUO+COL28-NC group. It is suggested that overexpression of COL28 can promote renal fibrosis. Tissue immunofluorescence confirmed that the UUO model with overexpressing COL28 was successfully constructed, and UUO itself could promote endogenous COL28 expression. Further, WB showed that overexpression of COL28 could promote *α*-SMA, Snail, and E-cadherin expressions in renal tissues, confirming that COL28 may aggravate renal interstitial fibrosis via promoting EMT.

Further, we investigated the molecular mechanism by which COL28 promotes proliferation and EMT in HK-2 cells. Our study examined transcriptome changes in HK-2 cells overexpressing COL28 by RNA-seq and screened a total of 286 upregulated DEGs and 158 downregulated DEGs. KEGG functional analysis showed that DEGs were mainly enriched in glycometabolism, glycolysis, glycogenesis, and so on. Studies have shown that abnormal glycolysis and glycometabolism are associated with tumor cell proliferation and EMT-related renal fibrosis [19, 25]. Combined with KEGG signaling pathway results, we also screened for HKDC1, a downstream regulator associated with COL28 promoting cell proliferation and EMT. Studies have shown that the HKDC1 is highly enriched in glucose metabolism, lipid metabolism, and protein metabolism pathways [26]. QRT-PCR also confirmed that HKDC1 was significantly upregulated in HK-2 cells overexpressing COL28. Recent studies indicate that HKDC1 has an oncogenic function in specific cancer types by different mechanisms, such as liver cancer [19], breast cancer [20], lymphoma [27], colorectal cancer [28], and lung cancer [29]. HKDC1 was proven to be substantially expressed in lung adenocarcinoma (LUAD) tissues and cell lines, and a correlation between HKDC1 positive expression with an aggressive phenotype and a poor prognosis in LUAD patients was found by Wang et al. HKDC1 overexpression accelerated glycolysis, proliferation, invasion, migration, EMT, and tumorigenicity, while knockdown of HKDC1 had the opposite functional consequences [30]. From the study of Chen et al., both clinical tumor tissues and breast cancer cells have high levels of HKDC1 expression. Through the SREBP1 binding motif on the HKDC1 promoter, PGC1 upregulates and coactivates HKDC1 expression. HKDC1 regulates metastasis, proliferation, apoptosis, and oxidative stress in breast cancer [20]. In their investigation of the potential mechanism of HKDC1 in the onset and progression of gastric cancer, Zhang et al. demonstrated that suppressing HKDC1 expression prevented the growth, invasion, and migration of gastric cancer cells as well as EMT [31].

Sterol regulatory element binding transcription protein 1 (SREBP1), a key homeostasis regulator, triggers adaptive responses to dietary/environmental stress, which may lead to lipid disturbances to maintain ideal membrane lipid composition and guarantee cell survival and function [32, 33]. As a major transcription factor, SREBP1 directly activates critical rate-limiting enzymes for cholesterol and fatty acids. Notably, cancer is assumed to be characterized by reprogramming of cell metabolism, and lipid metabolism is frequently disrupted in cancer cells to feed the biosynthetic demands of malignant behavior. Several studies have shown that the elevated SREBP1 expression was linked to poor prognosis of endometrial cancer, kidney cancer, liver cancer, breast cancer, and other solid tumors [34–36]. The study by Zhang et al. also suggested that increased SREBP1 expression could promote the proliferation and migration of breast tumor cells both in vivo and in vitro. Further, it was also shown that SREBP1 promoted tumor cell metastasis by inducing EMT, and its mechanism was to inhibit E-cadherin by forming coinhibitory complexes with HDAC1/2 and Snail, thereby regulating EMT [37].

Our study confirmed that HKDC1 and SREBP1 were expressed at higher levels of mRNA and protein in HK-2 cells when COL28 was overexpressed. The expression of HKDC1 and SREBP1 in UUO mouse renal tissues pretreated with COL28 adenovirus was also increased. By constructing the HKDC1 interfering agent, we infected the stable COL28 overexpression cell line with it and detected the effects of HKDC1 interfering on *α*-SMA and E-cadherin expressions. The findings indicated that interfering with HKDC1 expression could reverse the EMT caused by COL28 overexpression. It is suggested that HKDC1 is a downstream regulator of COL28 promoting EMT. Thus, in the future, assays determining the expression of COL28 could provide additional information on the kidneys' health and functions, and targeting the COL28/HKDC1 axis could be a potential treatment for personalized treatment.

## 5. Conclusions

In fibrotic renal tissue, expression of COL28 is significantly increased, and overexpression of COL28 further exacerbates renal fibrosis by promoting EMT through increased expression of SREBP1 and HKDC1. By targeting the COL28/HKDC1 signaling pathway, this provides a unique therapeutic approach for the treatment of renal fibrosis.

## Figures and Tables

**Table 1:** Quantitative real-time PCR primers.

Primer	Sequence 5′-3′	Length of primer (bp)	Length of product (bp)
HKDC1 F	GTTGCCCACCTTCGTCAGG	19	106
HKDC1 R	AGCGACTTGCACCTTCAGC	19	
SREBP1F	CGGAACCATCTTGGCAACAGT	21	141
SREBP1R	CGCTTCTCAATGGCGTTGT	19	
*β*-Actin F	GGGCCGGACTCGTCATAC	18	144
*β*-Actin R	GGGCCGGACTCGTCATAC	18	

**Table 2:** The top 15 high expression genes in the COL28-OE group compared to the COL28-NC group.

Gene	Type	log2 (fold_change)	*P* value
COL28	mRNA	13.209	<0.001
PAX2	mRNA	4.111	<0.001
HNF1B	mRNA	3.715	<0.001
PDZK1IP1	mRNA	3.286	<0.001
GPRC5C	mRNA	2.990	<0.001
TMEM240	mRNA	2.751	0.031
KLRC2	mRNA	2.734	0.046
HKDC1	mRNA	2.730	<0.001
TM4SF18	mRNA	2.608	<0.001
VWA5A	mRNA	2.600	<0.001
PTGIS	mRNA	2.556	<0.001
C3	mRNA	2.552	<0.001
EPGN	mRNA	2.537	<0.001
CX3CL1	mRNA	2.489	<0.001
FAM83A	mRNA	2.487	<0.001

**Table 3:** The top 15 low expression genes in the COL28-OE group compared to the COL28-NC group.

Gene	Type	log2 (fold_change)	*P* value
CD74	mRNA	-2.756	0.002
INHBB	mRNA	-2.651	<0.001
TMEM255A	mRNA	-2.628	<0.001
DAPK1	mRNA	-2.304	<0.001
REEP1	mRNA	-2.302	<0.001
CRYM	mRNA	-2.101	<0.001
ELAVL2	mRNA	-2.084	0.018
TIAM2	mRNA	-1.980	0.011
NEDD9	mRNA	-1.864	<0.001
FSTL1	mRNA	-1.821	<0.001
RNF144B	mRNA	-1.819	<0.001
CDK14	mRNA	-1.735	<0.001
C4ORF19	mRNA	-1.732	<0.001
LYPD6	mRNA	-1.723	<0.001
MEST	mRNA	-1.720	<0.001

## Data Availability

The corresponding author will provide access to the data used to support the findings of this study on request.

## References

[1] Xie Y., Bowe B., Mokdad A. H. (2018). Analysis of the global burden of disease study highlights the global, regional, and national trends of chronic kidney disease epidemiology from 1990 to 2016. *Kidney International*.

[2] Hewitson T. D. (2009). Renal tubulointerstitial fibrosis: common but never simple. *American Journal of Physiology. Renal Physiology*.

[3] Gewin L., Zent R., Pozzi A. (2017). Progression of chronic kidney disease: too much cellular talk causes damage. *Kidney International*.

[4] Rastaldi M. P. (2006). Epithelial-mesenchymal transition and its implications for the development of renal tubulointerstitial fibrosis. *Journal of Nephrology*.

[5] Kadler K. E., Baldock C., Bella J., Boot-Handford R. P. (2007). Collagens at a glance. *Journal of Cell Science*.

[6] Grimal S., Puech S., Wagener R., Ventéo S., Carroll P., Fichard-Carroll A. (2010). Collagen XXVIII is a distinctive component of the peripheral nervous system nodes of Ranvier and surrounds nonmyelinating glial cells. *Glia*.

[7] Reese-Petersen A. L., Willumsen N., Palau P. (2021). Evaluation of a novel biomarker of type XXVIII collagen formation, PRO-C28, in samples from cancer and heart failure with preserved ejection fraction patients. *Journal of Pharmaceutical and Biomedical Analysis*.

[8] Gebauer J. M., Kobbe B., Paulsson M., Wagener R. (2016). Structure, evolution and expression of collagen XXVIII: lessons from the zebrafish. *Matrix Biology*.

[9] Veit G., Kobbe B., Keene D. R., Paulsson M., Koch M., Wagener R. (2006). Collagen XXVIII, a novel von Willebrand factor A domain-containing protein with many imperfections in the collagenous domain. *The Journal of Biological Chemistry*.

[10] Schiller H. B., Fernandez I. E., Burgstaller G. (2015). Time- and compartment-resolved proteome profiling of the extracellular niche in lung injury and repair. *Molecular Systems Biology*.

[11] Pezzolesi M. G., Krolewski A. S. (2013). The genetic risk of kidney disease in type 2 diabetes. *The Medical Clinics of North America*.

[12] Ding S., Chen X., Shen K. (2020). Single-cell RNA sequencing in breast cancer: understanding tumor heterogeneity and paving roads to individualized therapy. *Cancer Communications*.

[13] Wang Z., Gerstein M., Snyder M. (2009). RNA-Seq: a revolutionary tool for transcriptomics. *Nature Reviews. Genetics*.

[14] Marioni J. C., Mason C. E., Mane S. M., Stephens M., Gilad Y. (2008). RNA-seq: an assessment of technical reproducibility and comparison with gene expression arrays. *Genome Research*.

[15] Khan M. W., Ding X., Cotler S. J., Clarke M., Layden B. T. (2018). Studies on the tissue localization of HKDC1, a putative novel fifth hexokinase, in humans. *Journal of Histochemistry & Cytochemistry*.

[16] Wilson J. E. (2003). Isozymes of mammalian hexokinase: structure, subcellular localization and metabolic function. *The Journal of Experimental Biology*.

[17] Guo C., Ludvik A. E., Arlotto M. E. (2015). Coordinated regulatory variation associated with gestational hyperglycaemia regulates expression of the novel hexokinase _HKDC1_. *Nature Communications*.

[18] Li G. H., Huang J. F. (2014). Inferring therapeutic targets from heterogeneous data: HKDC1 is a novel potential therapeutic target for cancer. *Bioinformatics*.

[19] Zhang Z., Huang S., Wang H. (2016). High expression of hexokinase domain containing 1 is associated with poor prognosis and aggressive phenotype in hepatocarcinoma. *Biochemical and Biophysical Research Communications*.

[20] Chen X., Lv Y., Sun Y. (2019). PGC1*β* regulates breast tumor growth and metastasis by SREBP1-mediated HKDC1 expression. *Frontiers in Oncology*.

[21] Song X., Lin N. H., Wang Y. L., Chen B., Wang H. X., Hu K. (2019). Comprehensive transcriptome analysis based on RNA sequencing identifies critical genes for lipopolysaccharide-induced epididymitis in a rat model. *Asian Journal of Andrology*.

[22] Wang X., Harris R. E., Bayston L. J., Ashe H. L. (2008). Type IV collagens regulate BMP signalling in _Drosophila_. *Nature*.

[23] Ortiz-Urda S., Garcia J., Green C. L. (2005). Type VII collagen is required for Ras-driven human epidermal tumorigenesis. *Science*.

[24] Yang H., Jin L., Sun X. (2019). A thirteen-gene set efficiently predicts the prognosis of glioblastoma. *Molecular Medicine Reports*.

[25] Li J., Liu H., Takagi S. (2020). Renal protective effects of empagliflozin via inhibition of EMT and aberrant glycolysis in proximal tubules. *JCI Insight*.

[26] Tian X., Zhang H., Zhao Y. (2018). Transcriptome analysis reveals the molecular mechanism of hepatic metabolism disorder caused by chromium poisoning in chickens. *Environmental Science and Pollution Research*.

[27] Chen Q., Feng J., Wu J. (2020). HKDC1 C-terminal based peptides inhibit extranodal natural killer/T-cell lymphoma by modulation of mitochondrial function and EBV suppression. *Leukemia*.

[28] Fuhr L., El-Athman R., Scrima R. (2018). The circadian clock regulates metabolic phenotype rewiring via HKDC1 and modulates tumor progression and drug response in colorectal cancer. *EBioMedicine*.

[29] Wang L., Yuan Li W., Fang Li F., Yan Yang C., Cai Mu D., Yong Zheng S. (2020). Bioinformatics analysis of HKDC1 expression in non-small cell lung cancer and its relationship to survival. *Journal of Nutritional Oncology*.

[30] Wang X., Shi B., Zhao Y. (2020). HKDC1 promotes the tumorigenesis and glycolysis in lung adenocarcinoma via regulating AMPK/mTOR signaling pathway. *Cancer Cell International*.

[31] Zuo-Yan W. L.- Z. H. A. N. G., Jia-Si M. A., Shan-Shan Z. H. E. N. G., Lei H. E., Chong Z. H. A. N. G., Ling-Hui Z. E. N. G. (2020). Effect of silencing hexokinase domain containing protein 1 expression on proliferation, invasion, metastasis and epithelial mesenchymal transformation of gastric cancer cells. *Chinese Journal of Pharmacology and Toxicology*.

[32] Hagen R. M., Rodriguez-Cuenca S., Vidal-Puig A. (2010). An allostatic control of membrane lipid composition by SREBP1. *FEBS Letters*.

[33] Wu D., Yang Y., Hou Y. (2022). Increased mitochondrial fission drives the reprogramming of fatty acid metabolism in hepatocellular carcinoma cells through suppression of Sirtuin 1. *Cancer Communications*.

[34] Lee J. H., Jeon Y. G., Lee K.-H. (2017). RNF20 suppresses tumorigenesis by inhibiting the SREBP1c-PTTG1 axis in kidney cancer. *Molecular and Cellular Biology*.

[35] Bao J., Zhu L., Zhu Q., Su J., Liu M., Huang W. (2016). SREBP-1 is an independent prognostic marker and promotes invasion and migration in breast cancer. *Oncology Letters*.

[36] Li C., Yang W., Zhang J. (2014). SREBP-1 has a prognostic role and contributes to invasion and metastasis in human hepatocellular carcinoma. *International Journal of Molecular Sciences*.

[37] Zhang N., Zhang H., Liu Y. (2019). SREBP1, targeted by miR-18a-5p, modulates epithelial-mesenchymal transition in breast cancer via forming a co-repressor complex with snail and HDAC1/2. *Cell Death & Differentiation*.

